# The underestimated fraction: diversity, challenges and novel insights into unicellular cyanobionts of lichens

**DOI:** 10.1093/ismeco/ycae069

**Published:** 2024-05-06

**Authors:** Patrick Jung, Laura Briegel-Williams, Burkhard Büdel, Matthias Schultz, Dennis J Nürnberg, Martin Grube, Paul M D’Agostino, Jan Kaštovský, Jan Mareš, Maike Lorenz, Manuel Luis Gil González, Manuela Dal Forno, Martin Westberg, Nathan Chrismas, Nicole Pietrasiak, Paul Whelan, Petr Dvořák, Alica Košuthová, Spyros Gkelis, Thorsten Bauersachs, Ulf Schiefelbein, Võ Thị Phi Giao, Michael Lakatos, INCb, International Network for research on unicellular CyanoBionts from lichens

**Affiliations:** Integrative Biotechnology, University of Applied Sciences Kaiserslautern, Pirmasens, Germany; Integrative Biotechnology, University of Applied Sciences Kaiserslautern, Pirmasens, Germany; Rhineland-Palatinate Technical University Kaiserslautern Landau, Kaiserslautern, Germany; Herbarium Hamburgense, Institute of Plant Science and Microbiology, University of Hamburg, Hamburg, Germany; Institute for Experimental Physics, Freie Universität Berlin, Berlin, Germany; Dahlem Centre of Plant Sciences, Freie Universität Berlin, Berlin Germany; Institute of Biology, University of Graz, Graz, Austria; Technical University Dresden, Chair of Technical Biochemistry, Dresden, Germany; Department of Botany, Faculty of Science, University of South Bohemia, České Budějovice, Czech Republic; Institute of Microbiology, The Czech Academy of Sciences, Třeboň, Czech Republic; University of Goettingen, SAG Goettingen, Goettingen, Germany; IES Tinajo, Lanzarote, Spain; Botanical Research Institute of Texas, United States; Museum of Evolution, Uppsala University, Uppsala, Sweden; Royal Botanic Garden Edinburgh, Edinburgh, UK; University of Nevada - Las Vegas, Las Vegas, United States; National Botanic Gardens, Ireland; Palacký University Olomouc, Olomouc, Czech Republic; Swedish Museum of Natural History, Sweden; Aristotle University of Thessaloniki, Thessaloniki, Greece; Institute of Organic Biogeochemistry in Geo-Systems, RWTH Aachen University, Aachen, Germany; University of Rostock, Botanical Garden, Rostock, Germany; VNUHCM–University of Science, Vietnam; Integrative Biotechnology, University of Applied Sciences Kaiserslautern, Pirmasens, Germany; International Network for research on unicellular CyanoBionts from lichens

**Keywords:** Chroococcidiopsis, Chroococcidiopsidales, Lichinales, cyanolichens, lichenology, phycology, photobiont, cyanobiont

## Abstract

Lichens are remarkable and classic examples of symbiotic organisms that have fascinated scientists for centuries. Yet, it has only been for a couple of decades that significant advances have focused on the diversity of their green algal and/or cyanobacterial photobionts. Cyanolichens, which contain cyanobacteria as their photosynthetic partner, include up to 10% of all known lichens and, as such, studies on their cyanobionts are much rarer compared to their green algal counterparts. For the unicellular cyanobionts, i.e. cyanobacteria that do not form filaments, these studies are even scarcer. Nonetheless, these currently include at least 10 different genera in the cosmopolitan lichen order Lichinales. An international consortium (International Network of CyanoBionts; INCb) will tackle this lack of knowledge. In this article, we discuss the status of current unicellular cyanobiont research, compare the taxonomic resolution of photobionts from cyanolichens with those of green algal lichens (chlorolichens), and give a roadmap of research on how to recondition the underestimated fraction of symbiotic unicellular cyanobacteria in lichens.

## Graphical Abstract

**Graphical Abstract:**
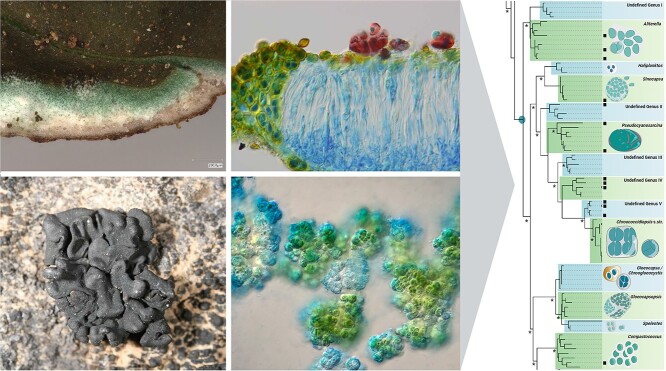

## Introduction

The symbiotic relationship between fungi and photosynthetic partners in lichens has fascinated biologists since 1867 when the Swiss botanist Simon Schwendener proposed his dual theory against the former consensus of an autonomous organism [1]. Since then, lichens have represented an iconic example of symbiosis due to the outdated idea that lichens are formed only by fungi with an algal partner [2]. The symbiosis is actually far more complex, involving many microbial organisms who significantly contribute to the association [e.g. 3–6]. Consequently, to reflect their complexity, lichens have been redefined as self-sustaining complex micro-ecosystems [7]. This has led to a new taxonomical framework for the main photosynthetic drivers within lichen symbiosis (i.e. the algae) in order to improve our understanding of their role in the association, for example, by dictating the ecophysiological capability of a lichen or their ecological niche. Fungi taking advantage of the photosynthetic ability of algae is considered to be one of the most successful nutritional stages among fungi (8), highlighting the overall importance of this specific symbiosis. Interestingly, the diversity of lichen-forming fungi exceeds 19 000 described species [8], but only about 200 species of photobionts, from a limited number of green algal and cyanobacterial genera, have formally been described [9–11]. Out of these lichen-forming fungi, about 90% share green algae as their main photobiont, which have been predominantly assigned to the genus *Trebouxia* (Trebouxiophyceae, Chlorophyta) [12], at least in temperate regions (besides *Asterochloris, Myrmecia, Trentepohlia*, and others [13]). *Trebouxia* has recently received a much-needed phylogeny-based taxonomic update [9], which is of major importance as the structure and diversity of lichen populations are mainly driven by abiotic factors, which limit them to certain habitats. This has often been linked to the ecophysiological properties of certain (*Trebouxia*) photobiont guilds since the mycobiont-photobiont pairing is not a random event [e.g. [13]]. Regarding cyanobacterial lichens, current knowledge suggests that photosynthetic activity of cyanobacteria strongly depends on the presence of liquid water. High air humidity alone cannot activate photosynthesis [e.g. [14]]. This restricts appropriate habitats for cyanolichens and may explain the limited number of cyanolichen species compared to the chlorolichens. Regardless of their dependence on liquid water, unicellular cyanobacterial lichens generally seem to appear in harsh environmental conditions such as inselbergs, deserts, and rocky seashores, which are physiologically stressful environments [15–19]. This suggests that the availability of appropriate microhabitats is key in distribution patterns, for example, numerous favorable xeric microhabitats are created by weathering of inselberg rock faces [18, 20]. Cyanolichens must be desiccation tolerant, and studies have shown that while free-living *Chroococcidiopsis* cells are damaged from desiccation events all symbiotic species survive desiccation damage free, these physiological interactions are not currently understood but the mycobiont evidently provides more than optimized CO_2_ and nutrient acquisition [21, 22].

For the most part, the known factors for determining lichen population structure are based on chlorolichens. Insights into cyanolichens, their mycobiont–cyanobiont relationship, and properly applied taxonomic treatments are rare and, thus, the degree of comparability between chlorobionts and cyanobionts is largely unknown. This not only has to do with different methodologies applied in phycology versus lichenology but also with an underestimation of cyanobiont diversity, which has recently been discussed in Jung et al. [23]. Cyanobiont diversity has mainly focused on cyanolichens that form a symbiosis with members of the filamentous, heterocytous cyanobacterial genera *Nostoc* [e.g. [24]] or *Rhizonema* [e.g. [10]]. Meanwhile, unicellular cyanobacteria have been little studied, although they have been identified as symbionts of lichens for decades [25, 26], including in intertidal lichens, where Pleurocapsales (*Hyella* spp.) have long been known as primary photobionts [27–29]. A broad community of researchers has acknowledged these issues, and the initial step to bring this underestimated fraction of unicellular cyanobacteria involved in lichen symbiosis into the spotlight has been the founding of an international consortium called INCb (International Network for CyanoBionts). In this article, we aim to detail the current knowledge, diversity and research outlook for unicellular cyanobionts and intend to highlight implications for other research disciplines.

## Why did we miss unicellular cyanobionts?

Elucidating and identifying species involved in symbiotic interactions is crucial for understanding highly integrated associations, which can be major factors determining ecological and evolutionary dynamics. Concerning lichens, Hoffman and Lendemer [30] estimated that 89.7% of all studies published between 2000 and 2016 involving molecular techniques focused solely on mycobionts, while a minority of studies additionally focused on the photobionts, they were limited to chlorolichens. Methodological difficulties when working on lichen photobionts are exacerbated during the process of algal isolation, which has resulted in only about 27 described *Trebouxia* species [31]. This is a surprisingly low number, since *Trebouxia* is the major lichen photobiont genus. Muggia et al. [9] recently presented an integrated taxonomic approach framing 109–113 candidate species distributed across four main *Trebouxia* lineages, this acts as a reference dataset for characterizing diversity in lichenized green algae.

**Figure 1:**
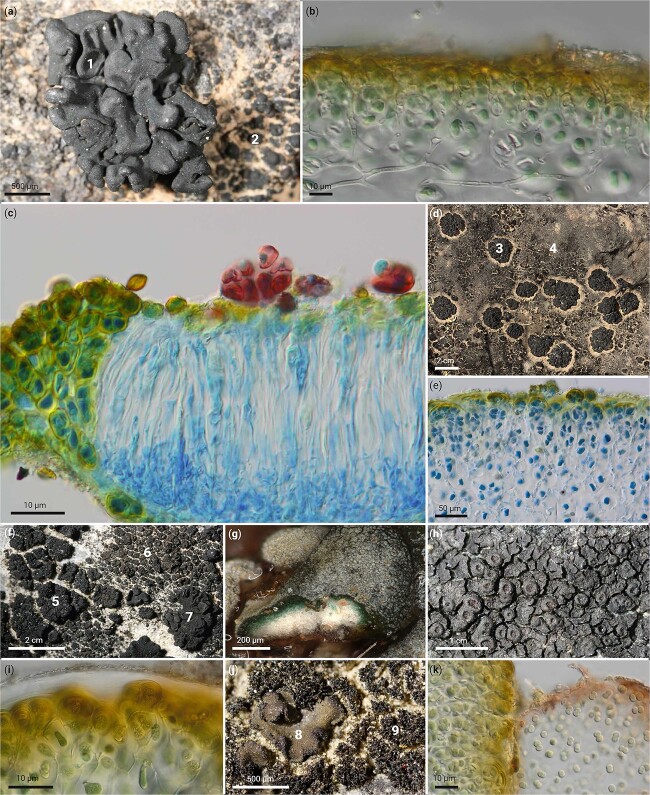
Cyanolichens and their unicellular cyanobacterial photobionts (cyanobionts). (a) Foliose *Lichinella iodopulchra* (1) and crustose *Pterygiopsis canariensis* (2) from Canary Islands. (b) Microscopic thin section of *Lichinella cribellifera* with unicellular cyanobionts. (c) Microscopic thin section of an apothecium of *Psorotichia* sp. from Mallorca stained with lactophenol blue showing lichenized unicellular cyanobacteria left of the apothecium and free-living unicellular cyanobacteria epiphytically on the hymenial disc (red). (d) Foliose *Paulia perforata* (3) together with other cyanolichens (4) from Dhofar, Oman. (e) Microscopic thin section of *Anema tumidulum* from Germany stained with lactophenol showing the unicellular cyanobionts. (f) Rosette forming *Anema tumidulum* (5), crustose *Psorotichia murorum* (9), and foliose *Lichinella schleicheri* (7) from Wallis, Switzerland. (g) Hydrated *Peltula* sp. from Australia with a cut thallus squamule showing the unicellular cyanobionts in the thallus. (h) Crustose, squamulose *Pterygiopsis concordatula* from Austria. (i) Microscopic thin section of *Paulia glomerata* from Switzerland. (j) Foliose *Peltula euploca* (8) and fruticose *Lichinella stipatula* (9) from Wallis, Switzerland. (k) Microscopic thin section of *Anema prodigulum* from Nevada (left) with epiphytic unicellular cyanobacteria (*cf*. *Aphanocapsa*; right).

For cyanobacteria the situation is very different. Most lichenological studies that consider cyanobionts focus on cyanolichens such as *Cora, Dictyonema, Erioderma, Leptogium, Lichina, Lathagrium, Pannaria*, or *Peltigera* with members of *Nostoc* or *Rhizonema* as the cyanobiont, two better-known filamentous genera with the ability to fix atmospheric nitrogen [e.g. [10, 24]]. Here, molecular data were obtained from genomic DNA extracts and—in most cases—without the isolation of the cyanobionts. Even if the isolation of the cyanobionts is intended, this is a highly complex process for several reasons:

(1) Nostocalean cyanobionts can be isolated by using nitrogen deficient media in order to diminish non-nitrogen fixing epiphytic algae or other cyanobacteria but DNA is still needed to confirm that it is the actual photobiont [23].
(2) Other free-living cyanobacteria may live associated with the lichens as epiphytes and to complicate matters further unicellular symbionts have been found to co-occur with the main filamentous cyanobiont. These have often been missed through Sanger sequencing and are only now becoming known through metabarcoding and metatranscriptomic technologies [15, 32].
(3) Free-living and lichenized nostocalean taxa cannot be identified to species level based on solely 16S rRNA molecular data due to high sequence similarity. Currently, only guilds can be captured based on the *rbcLX* gene region, which carries a much more limited set of reference sequences compared to the more frequently used full 16S rRNA or genome data [33].
(4) Even if cyanobionts can grow in culture without their symbiotic partner, the isolation of unicellular cyanobionts in particular is difficult because they are usually non-motile, forming small colonies with exceptionally slow growth rates [23]. Isolation is further complicated because cyanolichens with unicellular symbionts are tiny and often grow firmly attached to the substrate. This substrate, or the lichen itself, is often colonized by various other free-living cyanobacterial taxa [34] (Fig. 1), making the isolation process a tedious task with a high degree of uncertainty. This explains the lack of unicellular cyanobiont isolates which therefore makes morphological investigations impossible since the morphology of lichenized cyanobacteria and their isolated descendants can significantly differ [35]. Ultimately, this has led to several false assignments of cyanobacterial taxa involved in lichen symbiosis as these were based solely on microscopy. Based on morphological identifications of cyanobionts either in the lichen thallus or from cultured isolates, the following 10 unicellular genera have been described, although without specifying their symbiotic role: *Aphanocapsa*, *Chroococcus*, *Chroococcidiopsis*, *Cyanosarcina*, *Entophysalis*, *Gloeocapsa*, *Hormathonema*, *Hyella*, *Microcystis*, and *Myxosarcina*, but some of these identifications were rather doubtful (summarized in Büdel [26]). Some taxa can have complicated life cycles alongside morphological shifts so that confusion has historically distorted information on cyanobionts associated with certain lichens. An example is the lichen species *Gonohymenia* to which unicellular cyanobionts of the genus *Gloeocapsa* were assigned based on microscopy [36]. Cyanobionts of this lichen genus were again investigated by integrating morphological observations of isolates and 16S rRNA phylogenies, which resulted in the discovery of *Nostoc*-related filamentous cyanobacteria with complex life cycles involving almost-unicellular life-stages (“Zellvereinzelung” = separation of filaments into single cells, e.g. monocytes) [23, 37]. Untangling algal–fungal relationships is somewhat easier for chlorolichens since *Trebouxia* is almost exclusively found as a photobiont and shows well differentiated growth characteristics [9]; therefore, the photobiont is simpler to distinguish from other green algae during the isolation process.

Many lichenological studies focusing on photobionts now involve molecular data but unfortunately, they often rely only on universal cyanobacterial marker genes such as *rbcLX* or *trnL*, which have limited informative value and can lead to weak phylogenetic resolution [24, 38, 39]. As a result, lichenologists have created a molecular reference system that allows identification at the genus level but often does not correctly reflect their phylogenetic position. Fortunately, many new cyanobacterial genera can be designated by 16S rRNA sequences and unicellular cyanobiont species have been shown to produce phylogenies with higher levels of resolution than for *Nostoc* species [23].

Molecular data alone is not sufficient for describing cyanobacteria species or genera, so phycologists rely on the polyphasic approach, which was popularized in 2005 [40] and comprehensively reviewed in 2014 [41]. This method allows taxonomic classification of cyanobacteria by consolidating the phylogenetic position based on the full 16S rRNA and secondary structures of the ITS gene region with morphological observations, ecological factors, and biogeographical patterns. Genome based approaches are taking over but the 16S rRNA based approach is still standard for cyanobacteria and has recently undergone a major update so that 16S rRNA sequences, morphology and genome-derived data are congruent [42]. Additionally, the phylum has recently received its own curated database—CyanoSeq [43]. This allows more robust assignment of metabarcoding data to the latest taxonomic classification, including for unicellular photobionts that have been described. This is a major advancement since Silva, the predominantly used database, does not reflect the latest taxonomic status of the phylum [43].

**Figure 2:**
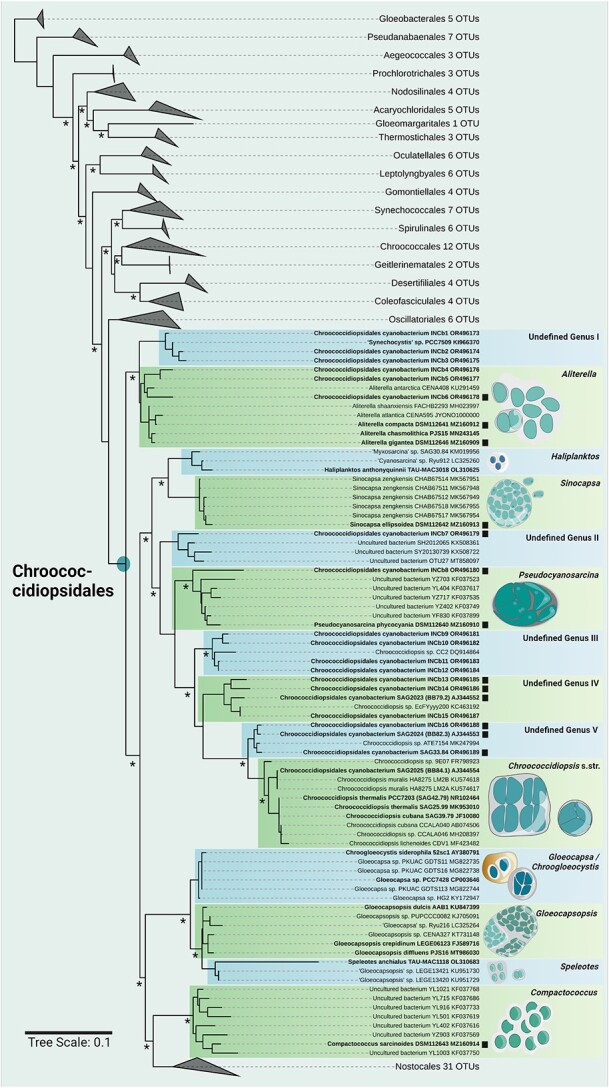
Maximum likelihood phylogenetic tree based on the full 16S rRNA gene sequences according to the latest phylogenetic update of Strunecký et al. (2023) [42]. This tree depicts the positioning of all cyanobacterial orders and gives details about the order Chroococcidiopsidales including illustrations of the most significant morphotypes. All sequences in bold are from isolates held by INCb. Sequences labeled with the strain identifier “INCb” were generated following the workflow described in [61] and the methodology therein was followed for the construction of the phylogenetic tree. Black squares indicate lichen symbionts (cyanobionts). All other sequences were derived from free-living isolates or uncultured cyanobacteria. Asterisks indicate >80% support derived from maximum likelihood and Bayesian inference.

Although the polyphasic approach is widely used, phycologists have rarely focused on cyanobacterial lichen photobionts. From molecular data we only know that the filamentous nostocalean genera *Nostoc, Macrochaete, Rhizonema, Rivularia, Scytonema, Stigonema,* and the unicellular genera *Chroococcidiopsis* and cf. *Pleurocapsa* are involved in cyanobacterial lichen symbiosis, although morphological studies, as stated above, have identified others. The genus *Chroococcidiopsis* has often been described as a major cyanobiont lineage [25] of various cyanolichens such as the genera *Anema, Peccania, Psorotichia*, and *Peltula* (all Lichinaceae) but has only been confirmed using molecular data by one study in 2002 for the lichens *Thyrea pulvinata, Anema nummularium*, and *Peltula euploca* [44]. Until recently, the genus name *Chroococcidiopsis* was assigned to cyanobacterial strains fulfilling the morphological characteristics that were redefined for the genus: cells divide by one to two binary fissions followed by many irregular simultaneous or succedaneous divisions and colonies have an irregular shape [45], but their taxonomic dissection has been hindered by the lack of sequenced reference strains. However, with the increasing wealth of strains isolated and assigned to *Chroococcidiopsis*, it became obvious that *Chroococcidiopsis* constituted a cosmopolitan, heterogenic and generalistic assemblage [46]. The establishment of the *Chroococcidiopsis sensu stricto* lineage (based on *Calothrix thermalis* PCC7203 (other strain identifier SAG42.79)) has enabled researchers to define the monophyletic family and order Chroococcidiopsidaceae and Chroococcidiopsidales, respectively [41, 45]. This phylogenetic benchmark has since encouraged researchers to define numerous new taxa (Fig. 2) and even taxonomic changes of traditional epilithic genera such as *Gloeocapsa* and *Gloeocapsopsis* are likely [47]. However, unicellular cyanolichens remained in obscurity until a recent investigation of seven lichens and their isolates led to the description of seven new cyanobacterial species, which formed two new unicellular genera (*Compactococcus* and *Phycocyania*) and assigned five cyanobacterial genera comprising symbionts—all of which were not previously known to have symbiont members [23]. Surprisingly, none of the isolated cyanobacteria turned out to be *Chroococcidiopsis*. The study was presented at the 22nd symposium of the International Association of Cyanophyte/Cyanobacteria Research in České Budějovice, Czech Republic, which demonstrated to the community that a large diversity of cyanobacteria involved in lichen symbiosis is waiting to be revealed.

## Where are we now?

INCb aims to shed light on the diversity of unicellular cyanobionts from lichens by isolating and characterizing them, creating a baseline to which additional aspects can be added in the future. Our ongoing investigations use the polyphasic approach combined with direct photobiont picking [48], with which we have isolated new unicellular cyanobionts and free-living cyanobacteria all from the orders Chroococcidiopsidales and Pleurocapsales. We can show that the few unicellular cyanobionts that have been identified cluster together with free-living cyanobacteria (Fig. 2). This provides evidence that the phylogenetic concepts for green algal photobionts, which have symbiont specific clades, and at least unicellular cyanobionts fundamentally differ. In addition, some genera within this unicellular order do not currently have symbiotic members based on DNA analysis, such as *Gloeocapsopsis*, *Gloeocapsa*, and *Chroococcidiopsis* sensu stricto, but this is likely biased by the current scarcity of research.

**Figure 3:**
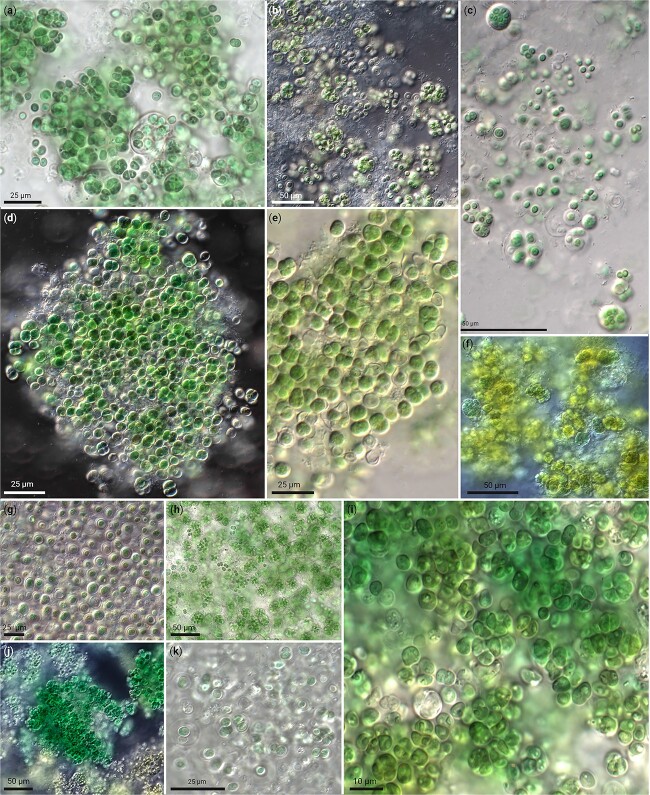
Free-living Chroococcidiopsidales isolates of the INCb consortium. (a) *Gloeocapsopsis dulcis* AAB1 from hypolithic biofilm of the Atacama Desert, Chile. (b, c) Chroococcidiopsidales cyanobacterium INCb10 from Israel (undefined genus III). (d, e) *Chroococcidiopsis cubana* SAG39.79 from soil in Cuba. (f) Chroococcidiopsidales cyanobacterium INCb4 (*Aliterella*). (g) Chroococcidiopsidales cyanobacterium INCb11 chasmoendolithic on granite from the Negev Desert, Israel (undefined genus III). (h) *Chroogloeocystis siderophila* CCAP1419 from an iron rich hot spring in Montana, USA. (i) *Gloeocapsa* sp. PCC7428 from a moderate hot spring in Amparai District, Maha Oya, Sri Lanka. (j) “*Synechocystis*” sp. PCC7509 from a rock in Schöllenen below Teufelsbrücke, Switzerland (undefined genus I). (k) *Chroococcidiopsis thermalis* SAG42.79 (other strain identifier PCC7203) from soil near Greifswald, Germany, the type strain of the order.

**Figure 4:**
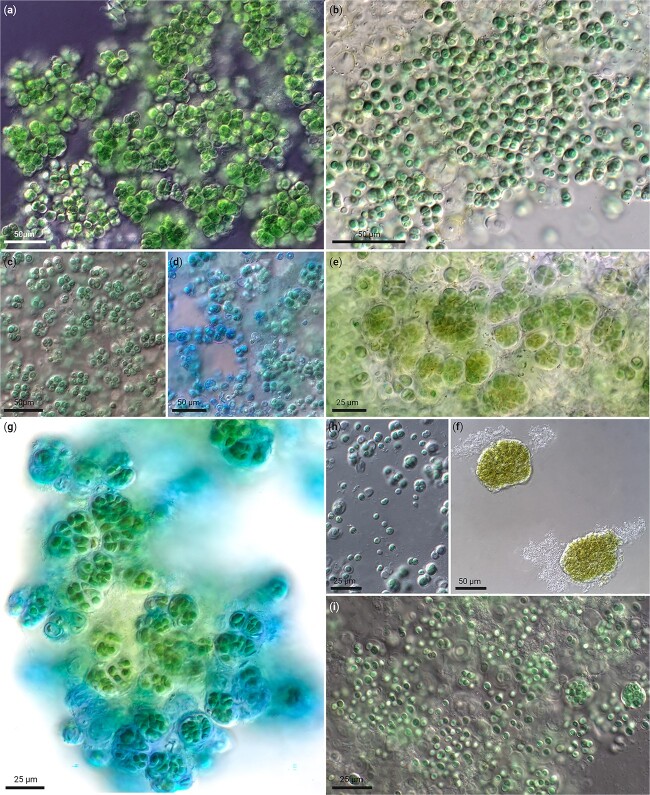
Lichenized Chroococcidiopsidales isolates of the INCb consortium. (a) Chroococcidiopsidales cyanobacterium SAG33.84 isolated from *Psorotichia columnaris* from Lanzarote, Spain (undefined genus V). (b) *Sinocapsa ellipsoidea* DSM 112642 isolated from *Peccania cerebriformis* from a rock near Graz, Austria. (c, d) *Pseudocyanosarcina phycocyania* DSM112640 isolated from *Peltula clavata* from seepage rock Queensland, Australia. (e, f) Chroococcidiopsidales cyanobacterium INCb16 isolated from *Lichinella* sp. from limestone near Huajuapax de Leon, Mexico (undefined genus V). (g) *Compactococcus sarcinoides* DSM 112643 isolated from *Gonohymenia* sp. from Australia. (h) Chroococcidiopsidales cyanobacterium SAG2024 isolated from *Anema nummularium var. nummulariellum* from Mexico. (i) Chroococcidiopsidales cyanobacterium INCb8 isolated from *Gonohymenia* sp. from Lanzarote, Spain (*Pseudocyanosarcina*).

Our preliminary data already indicates that there are many more undescribed and well-separated unicellular genera in the order Chroococcidiopsidales. These can be differentiated based on their 16S rRNA sequences, ecology and morphology (Fig. 2). Due to their unicellular appearance, most of these taxa have cryptic morphological features which made differentiating between free-living strains (Fig. 3) and lichenized strains impossible (Fig. 4) when relying only on microscopy. Additionally, unicellular cyanobionts of other orders cannot be excluded, as they are certainly not restricted to those we so far know, for example, recent research shows that lichen symbiotic members of the family Pleurocapsaceae may be more widespread than currently appreciated [see [15]].

## The research roadmap

The overall objective of the consortium is to connect lichenologists, microbiologists, and phycologists in order to promote the study of unicellular cyanobionts from lichens. We aim to gain new insights into the world of cyanolichens including information on both the photo- and the mycobionts, the holobiont’s host specificity, aspects of the symbiotic lifestyle, biogeographical patterns, and chemical ecology.

The isolates will be characterized based on their 16S rRNA sequences, morphology, ecology, genomics, and other potential metabolic characteristics. It will also be useful to create information on additional gene regions such as *rbcLX*, which will complement data so far generated by lichenologists, as this is a gene region that has frequently been amplified in previous studies. However, integrating molecular information from the lichen mycobionts using standard markers such as the nuITS, nuLSU, *RPB1, RPB2*, or mtSSU, will also provide new insights into the phylogenetic relationships of understudied lichens, such as those belonging to the Lichinales: *Peltula* [49] and/or *Lichina* [50]. Both genera have recently been identified as part of an ancient lineage of symbiotic Ascomycetes now called Lichinomycetes dating back 300 million years, designating them as the common ancestor of Eurotiomycetes and Lecanoromycetes [51].

A result of the difficulties in cyanobionts specimen collection and isolation is their underrepresentation at the genome level. In NCBI, a total of 24 lichen cyanobiont genomes are currently available compared to 4506 cyanobacterial genomes (20 July 2023). Of the 24 cyanobiont genomes, 21 belong to *Nostoc* spp., with the remainder belonging to *Rhizonema* spp*.* [52]. However, the first attempts based on full genome data of free-living and lichenized *Nostoc* strains identified genes potentially involved in symbioses, which may help to improve taxonomic resolution [53]. It would not be surprising for phylogenies with whole-genome sequencing based on ~100 core genes to become as common as 16S rRNA sequencing in the future. The increased isolation efforts of INCb will expand the genomic data, and result in the first publicly available unicellular cyanobiont genome. Rapidly growing numbers of genome sequences of cyanobacteria provide an increasing amount of evidence that single genetic markers, several markers, or morphological data have only limited power when recognizing the diversity between and within species [52, 54]. Additionally, it is likely that one lichen thallus can host several lineages of cyanobionts due to the potentially huge diversity at the genome level within single lineages of cyanobacteria [e.g. [55]].

Furthermore, metagenomics and metabarcoding will play important roles for unravelling uncultivatable cyanobiont diversity whilst also shedding light on the entire symbiotic consortium. Genomic data will additionally promote studies on detection of novel metabolic pathways, novel genes, and thus interesting compounds for biotechnological purposes. Cyanobacterial metabolomics is difficult due to the complex composition of the metabolome [56]; therefore, whole-genome sequencing provides an alternative approach for the discovery of novel molecules and biosynthetic enzymes from symbiotic cyanobacteria [57].

Cyanolichen investigations are thus a crucial focus for virtually any-omic technique, especially given the large herbarium collections of lichens worldwide, which provide a substantial source of historical data from various geographical locations. It has already been shown that DNA of herbarium lichen specimens can be amplified [58], some even being over a hundred years old [59], offering the possibility to investigate both present and historical diversity and interactions patterns.

Beyond phylogeny and evolution, there are important questions to be asked about the functional ecology of unicellular cyanobacteria in lichen symbiosis. Photobiont selection is fundamentally linked to the ecology of lichenized fungi and how unicellular cyanobacteria fit into this needs to be further elucidated. What physiological interactions exist between unicellular cyanobacteria and lichen fungi? Under what circumstances do associations with unicellular lineages develop alongside, or in preference to, associations with co-occurring filamentous cyanobacteria? Adaptions of photobionts, either unicellular or otherwise, may be as broad and diverse as the environments the lichens inhabit and multi-faceted studies that combine community, genomic, transcriptomic, and lipidomic approaches together with ecophysiology and environmental factors offer vast potential for investigating the details of these interactions.

## Future outlook

The greatest obstacle to overcome when investigating the diversity of unicellular cyanobacterial symbionts of lichens is the fact that—so far—most symbiotic cyanobacteria can be found free-living next to the lichens. This is common knowledge for cyanobacteria-lichen symbioses, but recent evidence has shown that at least 80% of all green algal genera involved in lichen symbioses also occur free-living [60]. This, along with the small size of most cyanolichens, highlights the requirement for methods that allow an accurate differentiation between lichen photobionts and their free-living members that are often adhered to the substrate or epiphytically to the lichen. Such a method, using a direct PCR technique on a small scale has recently been introduced [61], which could, in the future, be complemented by a culture attempt based on a micromanipulator that allows picking of single cells directly from inner parts of lichens comparable with the technique described in [62]. The direct PCR method which requires low biomass inserts has been shown to be suitable for the target organisms because all sequences of strains labeled with “INCb” in Fig. 2 were generated using the workflow as described in [61].

In order to generate an initial overview of the diversity of the unicellular lichen cyanobionts it might be helpful to start with a selection of lichen specimens of which a phylogenetic backbone already exists such as the one for the genus *Peltula* [63]. Such a curated specimen collection could then act as the basis for the identification of the cyanobionts using the methods mentioned above. As a future perspective, two scenarios can be speculated upon: (i) the species concept and state-of-the-art methodology to generate a cyanobiont phylogeny according to Strunecký et al. [42] helps to describe new genera and species with mostly cryptic morphological features as depicted in Fig. 2. This will be in line with the general taxonomic system currently applied for cyanobacteria but might lead to low phylogenetic resolutions comparable to what we know about symbiotic *Nostoc* spp. [e.g. [24]]; or (ii) a new taxonomic concept for cyanobacterial symbionts might be applied, similar to the lineage-based system introduced for *Trebouxia* spp. including the designation of species candidates [8]. Consequently, this new system must then be based on new or additional genetic markers or even full genomes and a shifted weight put on certain morphological features comparable with the chloroplast structure of *Trebouxia* spp. [64]. Currently, this might be speculative, but it frames a future outline that can be discussed by the research community working on cyanobacterial symbionts. We hope that this article contributes to the recently increasing interest in lichen symbionts highlighted by insightful novel perspectives on the topic [8, 11, 60] by adding the underestimated fraction of unicellular cyanobacterial lichen symbionts to the spectrum.

## Data Availability

All generated DNA sequences were submitted to (NCBI) GenBank under the accession numbers indicated in the phylogenetic tree (Fig. 2).
